# Arthroscopic Tenotomy of the Long Head of the Biceps Tendon and Section of the Anterior Joint Capsule Produce Moderate Osteoarthritic Changes in an Experimental Sheep Model

**DOI:** 10.3390/ijerph18147471

**Published:** 2021-07-13

**Authors:** Umile Giuseppe Longo, Francisco Forriol, Vincenzo Candela, Salvatore Maria Tecce, Sergio De Salvatore, Jose R. Altonaga, Andrew L. Wallace, Vincenzo Denaro

**Affiliations:** 1 Department of Orthopaedic and Trauma Surgery, Campus Bio-Medico University, Via Alvaro del Portillo, 200, Trigoria, 00128 Rome, Italy; v.candela@unicampus.it (V.C.); salvatoremaria.tecce@alcampus.it (S.M.T.); s.desalvatore@unicampus.it (S.D.S.); denaro@unicampus.it (V.D.); 2 Orthopaedic Surgery Department, Hospital Universitario Puerta de Hierro, Majadahonda, 28222 Madrid, Spain; fforriol@mac.com; 3 Surgery Department, Facultad de Veterinaria Universidad de Leon, 24071 León, Spain; jarodma@unileon.es (J.R.A.); wallace@fortiusclinic.com (A.L.W.)

**Keywords:** shoulder, osteoarthritis, surgery, animal, model

## Abstract

Osteoarthritis (OA) of the glenohumeral (GH) joint is a common cause of shoulder pain, resulting in considerable invalidity. Unfortunately, the study of its pathogenesis is challenging. Models of OA are necessary to identify specific targets for therapy and to be able to interfere with the development and evolution of OA. This study aims to assess the effect of an arthroscopic tenotomy of the long head of the biceps tendon (LHBT) and section of the anterior glenohumeral joint capsule on the ovine glenohumeral joint. In addition, the authors aim to validate and evaluate the reliability of a modified semi-quantitative MRI score to assess joint degeneration in a sheep’s shoulder. Eight skeletally mature sheep received an arthroscopic tenotomy of the LHBT and section of the anterior joint capsule and were euthanized four months after surgery. All animals tolerated the surgery well, and no complication was recorded for six weeks. Moderate degenerative changes to the ovine shoulder joint were found on MRI and histological evaluation. The arthroscopic tenotomy of the LHBT and the anterior glenohumeral joint capsule section caused moderate degenerative changes to the ovine shoulder joint.

## 1. Introduction

Osteoarthritis (OA) consists of a structural breakdown of cartilage and bone and causes chronic disability worldwide, affecting millions of people [1]. OA of the glenohumeral (GH) joint is a frequent cause of shoulder pain, resulting in considerable disability [2,3]. To date, no effective interventions have been developed, except for joint arthroplasty in end-stage disease [4]. The study of GH-OA pathogenesis is not easy. Human joint specimens can be obtained only during joint arthroplasty or arthroscopy. OA animal models are needed to understand OA pathophysiology and identify targets for treatment. OA animal models can be surgically or chemically induced. However, animal models are not always reproducible in humans. For example, in animals, anterior cruciate ligament (ACL) transection or meniscectomy produces a faster and worse OA than humans [5,6]. Although several models of surgically induced destabilization of the knee joint are available, few studies have evaluated the degenerative changes in the articular cartilages of the shoulder in surgical models of joint destabilization [7]. To date, the absence of validated animal models to study shoulder OA is a limitation for research [8]. Tendon transection and denervation in a small animal model have been used. Reuther and colleagues observed glenohumeral OA changes in rat joints after open massive rotator cuff tears repair [9]. However, the open approach is related to the high risk of tissue damage. The arthroscopic approach has never previously been used to determine an experimental model of shoulder OA. Moreover, data on the pathophysiology of articular cartilage changes in experimental shoulder OA are limited to small animal models [10]. No data on large size animals are available. The sheep shoulder has never been evaluated as a model for shoulder OA. The use of skeletally mature sheep has several advantages in experimental settings [11]. Sheep are convenient animal models for the ease of handling, limited management costs and societal acceptance as research animals [12]. During functional motions, both static (ligaments, joint capsule and bony anatomy) and dynamic structures provide congruency and stability to the shoulder [13]. Increased translation of the humeral head and consequent mechanical instability could be produced by disrupting the shoulder balance.

Furthermore, the precise role of the biceps is still discussed [14]. This tendon probably contributes to joint stability, but the exact mechanism of action remains unclear [15,16,17,18]. Some authors described only a moderate effect on shoulder stability, while others sustained its role in humeral head depression (working with the supraspinatus) [7,8,11]. Clinicians well know the pathologies of the long head of the biceps tendon (LHBT); however, the proper treatment for persistent biceps tendinopathy is still debated [19]. The most common treatments for persistent LHBT tendinopathies are tenotomy and tenodesis (open or arthroscopic). Zabrzyński and colleagues reported significant improvements in clinical outcomes after LHBT tenotomy in the long-term follow-up [20]. However, the exact effect of this specific approach is still unclear due to the complexity of the shoulder injuries [20]. LHBT tenotomy is commonly performed in shoulder arthroscopy, but if an anterior capsule section is also performed, there is a high risk of shoulder OA [20].

This study hypothesised that a surgical destabilization of the anterior part of the GH joint and an arthroscopic tenotomy of the long head of the biceps tendon (LHBT) would lead to mechanical instability and, consequently, OA in a large-animal model.

This study aims to evaluate the histological and radiographical effects of a surgical destabilization of the anterior part of the GH joint and an arthroscopic tenotomy of the LHBT. The second aim of the study is to validate and assess the reliability of a semi-quantitative modified magnetic resonance imaging (MRI) score to grade the degenerative joint modifications in the animal’s shoulder (sMOASS—Sheep MRI Osteoarthritis Shoulder Score).

## 2. Materials and Methods

### 2.1. Study Design

The ARRIVE guidelines for the reporting of animal experiments were followed in the present study. Sheep were obtained from the Veterinary Department of Leon University. The owner of the animals signed written informed consent. Eight sheep were used for the study. According to indications of Osteoarthritis Research Society International (OARSI) [21], only “skeletally mature” (≥2 years of age) animals were used. Sheep were held in individual cages and were fed with a standard diet and water ad libitum. No animal presented any health problems. Sheep were accommodated in a temperature-controlled room (23 °C) with a standard 12-h dark\light cycle (6:00 p.m.–6:00 a.m.).

Arthroscopic tenotomy of the LHBT and section of the anterior joint capsule were performed in sheep. Four months after surgery, a lethal injection of phenobarbitone was used to euthanize the sheep. Histological examinations and MRI throughout the GH joints of all animals were performed [22].

### 2.2. Surgical Procedure

Animals were handled in conformity with current animal protection laws.

Thiopental (1–2 mg/Kg), atropine (0.5 mg/Kg), ketamine (5 mg/Kg) and fentanyl (0.005 mg/Kg) were used to induce the general anaesthesia. Halothane (1%), O_2_ (40%) and fentanyl (30 min/each) were used to maintain the anaesthesia after the intubation. Cefazolin sodium (1 g/intravenous) was administered at induction, midoperatively and at the end of the procedure. The post-operative antibiotic protocol included 1 daily administration of procaine penicillin (3,000,000 units/subcutaneous) per 5 days.

**Figure 1:**
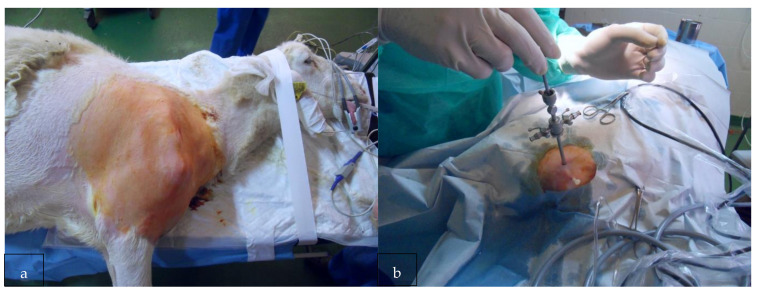
(**a**) Shoulder arthroscopy was performed in the lateral decubitus with standard portals. (**b**) Standard portals were performed (posterior and antero-lateral).

**Figure 2:**
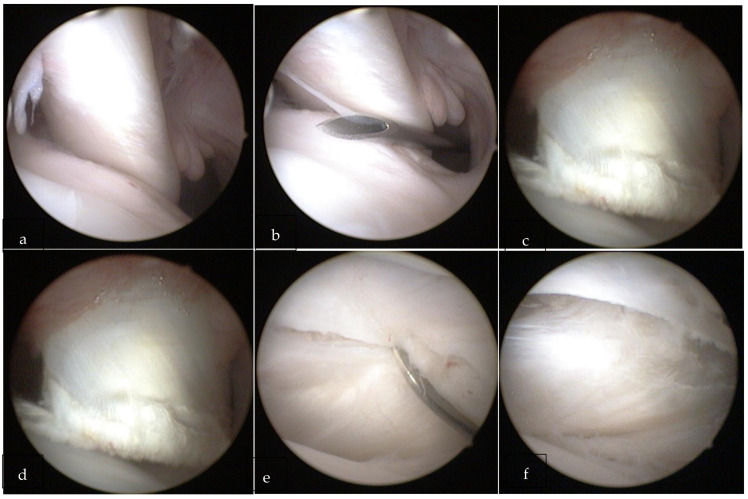
(**a**) Posterior view of the right shoulder showing the intact long head of the biceps tendon. (**b**) A standard anterior approach was established through a needle. (**c**) Tenotomy of the long head of the biceps tendon was performed. (**d**) Section of the anterior joint capsule was performed. (**e**,**f**) Section of the anterior joint capsule.

The same surgeon performed all surgical interventions in the lateral decubitus on the same operative day. Lesions of the GH joint of the right shoulder were performed for each sheep (Figure 1). After a diagnostic arthroscopy through standard portals (posterior and antero-lateral), the tenotomy of the LHBT and transverse section of the anterior joint capsule (Figure 2a–f) were performed. After surgery, weight-bearing was allowed in all the sheep. The wounds were not cleaned as they had healed without any problem in a few days.

### 2.3. Imaging

All sheep underwent magnetic resonance under sedation the day before surgery and 16 weeks after surgery. The same sedation protocol of the surgery was used to perform the MRI. 3T MRI system (Siemens Trio, Erlangen, Germany) was used. The entire shoulder was scanned in all three planes. T1 and T2 fat saturation images were obtained in alignment with the GH joint. The imaging protocol comprised sagittal spin-echo proton density, T1-weighted images (3 mm slice thickness and a 256 × 192 pixel matrix), T2-weighted images (3 mm slice thickness and a 256 × 192 pixel matrix), coronal and axial fat-suppressed spin-echo proton density.

Images were evaluated using Osirix software (Pixmeo Geneva, Switzerland, version 12.0). Two researchers with extensive orthopaedic imaging experience analysed all the images in a blinded manner.

We adapted the sheep MRI Osteoarthritis Knee Score (MOAKS), a semi-quantitative score [23] that evaluates cartilage and bone marrow lesions (BML) in the knee. BML included cyst, osteophytes, synovitis and effusion. The main alterations comprised: a modified sub-regional delimitation (the glenoid and humeral articular surface were divided into four sub-regions) and a different system for quantifying degenerative changes. We changed the suffix “K”, which refers to the knee, to adapt this scale to shoulder sMOASS (Sheep MRI Osteoarthritis Shoulder Score).

The glenoid articular surface was divided into 4 regions: anterosuperior and anteroinferior glenoid (ASG and AIG), posterosuperior and posteroinferior glenoid (PSG and PIG). The humeral articular surface was divided into four regions: anterior-superior and anterior-inferior humerus (ASH and AIH), posterior-superior and posterior-inferior humerus (PSH and PIH). Therefore, the glenoid articular surface included regions ASG, AIG, PSG and PIG and the humeral articular surface comprised regions ASH, AIH, PSH and PIH.

Articular cartilage (AC) was evaluated using fat-suppressed T2-weighted FSE images. Size of any cartilage loss was evaluated (0: none, 1: <10% of region of cartilage surface area (CSA), 2: 10–75% of region of CSA, 3: >75% of CSA). The maximum cartilage scores for glenoid and humerus (4 sub-regions with 3 points/sub-region) and the whole shoulder were: 12, 12 and 24, respectively. BMLs were evaluated on fat-suppressed T2-weighted FSE images and were recognized as areas with higher signal intensity. They were classified by size related to the total volume of the sub-region occupied by BML in each of the two surface regions (0: none, 1: <33% of sub-regional volume, 2: 33–66% of sub-regional volume, 3: >66% of sub-regional volume). The maximum BMLs score for the glenoid and humerus and the entire shoulder were 12, 12 and 24, respectively. The size of BML was used to classify them: 0: none, 1: small (<10% of the corresponding sub-region size), 2: medium (10–20% of the corresponding sub-region size), and 3: large (>20% of the corresponding sub-region size). Effusion was evaluated on the axial view on T2/PD/IW-w fat-suppressed sequences as a fluid equivalent signal within the joint cavity. The effusion was classified as normal (0), mild (1), moderate (2) and severe (3). Maximum scores for the entire shoulder were 3. A maximum score of 75 points, equivalent to 100% of shoulder joint degeneration, was developed combining the previous scores. Two researchers, within 16 weeks, performed two independent evaluations in a blinded manner.

### 2.4. Shoulder Arthritis According to the Histopathologic Scoring System

Sheep were sacrificed 16 weeks post-operative. The contralateral limb was used as control. After euthanisation, the treated joint and the control joint were dissected and prepared for macroscopic and histological assessment. Shoulders were acquired en bloc and fixed in 10% neutral buffered formalin (at 20° Celsius) for five days. Then, shoulders were decalcified for 21 days in 10% w/v EDTA and finally placed in paraffin. Shoulders were cut using a diamond saw. Three specimens from each group were evaluated by two separate blinded observers who performed the quantitative histomorphometry. The sections were placed on positively charged glass slides at 60 °C and de-paraffinized in xylene. Ethanol was used to rehydrate the section. Alcian blue and Safranin O were used. A Leica DFC320 light microscope was used with a 10× magnification. A scoring system, based on the Murine Shoulder Arthritis Score (MSAS) classification system described by Zingman [24], for evaluating the sheep shoulder’s degeneration following tenotomy of the LHBT and section of the anterior joint capsule was developed. The suffix “M”, which refers to murine, was changed to “O” to adapt this scale to the sheep shoulder (OSAS, Ovine Shoulder Arthritis Score). The final OSAS scores were classified and reported in Table 1. Each reviewer quantified all the parameters included in the OSAS score.

### 2.5. Statistical Analysis

Continuous outcomes measures were reported using means and standard deviations. The number of animals needed between groups was determined through an a priori power analysis. Using an Effect Size (ES) of 1.2, with an alpha value of 0.05 and a beta value of 0.08, eight animals were required in each group. A percent agreement between raters was performed to determine the agreement between observers in the OSAS. The Shapiro–Wilk test was used for testing normality. Wilcoxon Signed Ranks Test determined whether significant differences existed among groups. Significance was determined as *p* < 0.05.

## 3. Results

All animals tolerated the surgery well, and no complications were recorded for 16 weeks. During surgery, no macroscopic anomalies were noted in the animals.

### 3.1. Pre-Operative MRI Evaluation

**Figure 3:**
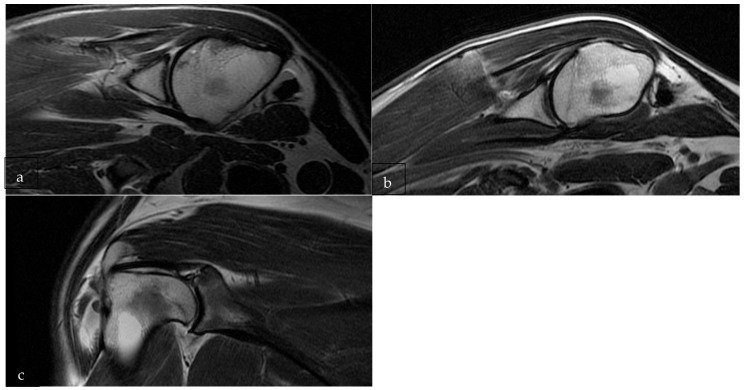
(**a**–**c**) Loss of cartilage surface area in the glenoid. (**b**,**c**): BML of the superior aspect of the glenoid.

The average cartilage score for all of the shoulders was: 3.25 (+/−1) (Figure 3a). The average BMLs score for all the shoulders was 3 (+/−1.5) (Figure 3b,c). The average osteophytes score was 4.25 (+/−1.8). Finally the average effusion score was 0.66 (+/−0.8).

### 3.2. Post-Operative MRI Evaluation

The average cartilage score for all the shoulders was: 3.37 (+/−0.8). The average BMLs score for all the shoulders was 3.37 (+/−1.6). The average osteophytes score was 4.38 (+/−1.9). Finally, the average effusion score was 0.66 (+/−0.8). Moderate degenerative changes to the ovine shoulder joint were found. Degenerative changes were observed by analysing the post-operative MRI of the ovine GH joints. However, significant differences were not found by comparing the score assigned to the individual areas. Moreover, no significant differences between the pre- and post-operative MRI scores were reported (Table 2).

The interobserver agreement between “Observer 1” and “Observer 2” regarding the pre-operative MRI evaluation was 79.5%. On the other hand, the interobserver agreement between “Observer 1” and “Observer 2” regarding the pre-operative MRI evaluation was 83.5%. The correlation between pre-operative MRI and post-operative MRI was 0.9736 for “Observer 1” and 0.9912 for “Observer 2”.

### 3.3. Histological Assessment

**Figure 4:**
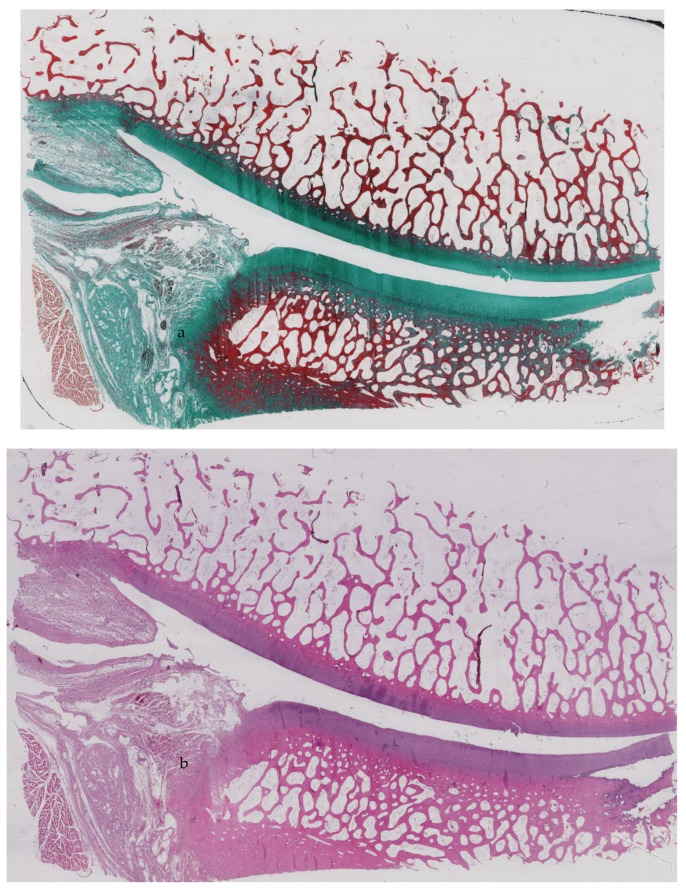
(**a**,**b**) Histology of the glenohumeral joint.

Histological assessment at 16 weeks showed a proliferative phase of the cartilage with a double layer superficial to the tidemark. Moreover, a superficial layer filled with mitotic figures was present. The humeral head was filled more by bone marrow than trabecular bone (Figure 4a,b).

For the injured shoulder, the average sphericity of articular portion of humeral head score was: 1.25 (+/−0.2). The average cellularity score was 1.87 (+/−0.9). The average subchondral bone morphology score was 2 (+/−0.3). The average alcian blue staining score was 1.37 (+/−0.4). Finally, the average pannus score was 0 (+/−0).

For the control shoulder, the average sphericity of articular portion of humeral head score was: 1.12 (+/−0.2). The average cellularity score was 1.75 (+/−0.7). The average subchondral bone morphology score was 1.87 (+/−0.2). The average alcian blue staining score was 1.25 (+/−0.5). Finally the average pannus score was 0 (+/−0).

No statistically significant differences were found between the OSAS score of the operated joint and the contralateral control.

## 4. Discussion

The most relevant finding of this work is that the arthroscopic tenotomy of the LHBT and section of the anterior GH joint capsule cause moderate degenerative changes to the ovine shoulder joint.

The LHBT tendinopathy is a relevant problem in orthopaedics as disorders of the LHBT are recognized as a common source of shoulder pain and disability [25]. LHBT disorders could be divided into three main groups (despite various classifications) according to the aetiology: inflammatory, instability and trauma [26,27]. Moreover, LHBT disorders could be frequently associated with rotator cuff tears [25].

OA development is not a linear process. The type of animal model, the conditions of the stables and cages may influence the results of the experiments [28]. To prevent bias and improve experimental rigour, the Animals in Research Reporting In Vivo Experiments (ARRIVE) guidelines were adopted and followed before the onset of experiments [29]. To increase the reliability of data interpretation and reproducibility, variation between experiments was minimised.

The primary outcome measure was MRI changes evaluated by the sMOASS scale. The secondary outcome was alteration at the histopathological analysis as evaluated by the OSAS scale.

All the sheep were collected from the same place to decrease the bias due to animal status (stress of shipping, different diet and microbiome). All the selected outcomes were compatible with each other, therefore not affecting sample size and study design. Sampling sites were pre-specified and standardised. The use of a single surgeon represents a strength point of this study. All surgeries were performed the same day, avoiding changes in temperature, humidity, food, water, noise/vibrations, staff, facility disease status, light cycles, etc. A different person performed animal identification to avoid bias. Each histopathological slide was scored twice in a blind fashion and a randomised order using validated scoring systems. Furthermore, imaging analysis was performed twice in a blind fashion and evaluated in a randomised order by two blinded researchers according to a pre-specified protocol [29]. The differences between sheep would be severely influenced by the animals’ ages, resulting in different conclusions regarding the OA process investigated [28]. For this reason, all the animals included in the present study were the same age.

The present paper assumes that LHBT tenotomy could result in OA development. However, this is not necessarily true for human patients. In early shoulder OA or rotator cuff injuries, LHBT tenotomy may provide significant results. Arthroscopic tenotomy does not seem to alter the progressive degenerative radiographic changes that occur with long-standing rotator cuff tears [30]. Moreover, LHBT tenotomy is a common and valid treatment for chronic tendinopathy [31].

The present study has some limitations. Firstly, the ovine animal model had differences in joint anatomy and mechanics compared with humans. To be specific, the range of motion of the human shoulder is wider compared to sheep [32]. Lastly, Sonnabend and colleagues reported that the tendons of the supraspinatus, infraspinatus and teres minor muscles share a common insertion site in humans. Instead, in sheep, these tendons are inserted independently [33].

Weight-bearing was allowed in all the animals after the surgery. The effect of weight on joint degradation is unknown. In addition, sheep are quadrupeds and therefore have intrinsically different joint loading mechanisms. A semi-quantitative score was used to assess the cartilage status in the present study [34]. The limitations of these systems are the categorizations in classes, according to the ICRS Committee [35]. The ICRS score is a useful histological comparison tool that employs a visual scale. With this method, the researcher can rate the sample by matching its characteristics to a graduated board of pictures. In the case of regenerated tissue, the observer assigns a score of 3. Conversely, in inadequately regenerated tissue, a score of 0 is attributed [35]. The sheep model was employed as it is very similar to the human model. Cartilage and glenoid wear, joint space narrowing, humerus elevation and humeral head collapse present similar characteristics to humans. Previously, other animal models (dog, mouse, rabbit and guinea pig) have been employed, reproducing human-like injuries (rupture of the anterior cruciate ligament, meniscectomy and other combined injuries) [36]. The a priori power analysis of the sample size was regulated to allow verification of gross differences. Therefore, the small sample size will not provide reliable data supporting good inter- and intraobserver differences. Further studies with a higher sample size are required to confirm the results. Lastly, the GH joint capsule section and the tenotomy of the LHBT will not result in severe OA.

The arthroscopic tenotomy of the LHBT and section of the anterior GH joint capsule was never used before, to the best of our knowledge. The joint instability leads to an altered mechanical load that could initiate osteoarthritis [36]. Previous animal studies showed that supraspinatus and infraspinatus detachment leads to reduced joint movements [24]. The mechanical and anatomical properties of the glenoid cartilage and the joint functionality may be reduced in cases of massive injuries of the supra- and infraspinatus [24]. These data support studies suggesting that joint instability caused by impaired loading can lead to cartilage damage [37,38].

## 5. Conclusions

The arthroscopic-induced instability protocol adopted in the present study to induce experimental osteoarthritis of the shoulder in an in vivo sheep model provided moderate degenerative osteoarthritic changes. With the animal model described in the present paper, it could be possible to produce OA and study the degenerative changes of cartilage. Further translational research that uses this model could provide new information on the aetiology of and the possible therapies for human OA.

## Figures and Tables

**Table 1:** Shoulder osteoarthritis according to the histopathologic scoring system. The table was adapted from the study by Zingman et al. [24].

Points	0	1	2	3
**Sphericity of articular portion of humeral head**	100% spherical	80% spherical	50% spherical	Mostlyflattened/pitted
**Glenoid bony erosion**	0	<20%	>20%, <50%	>50%
**Cellularity**	Obvious hypocellular layer superficial to tidemark	Reactive cellular layer superficial to tidemark	Scant layer superficial to tidemark	Tidemark difficult to distinguish/ inconsistent
**Subchondral bone morphology**	Well trabeculated subchondral bone without bone marrow close to osteochondral junction	Scant bone marrow approaching osteochondral junction	<50% of subchondral bone infiltrated by marrow space	>50% of subchondral bone infiltrated by marrow space
**Alcian blue staining**	Intense blue staining deep to tidemark	Two separate areas of intense blue staining: one deep to tidemark and one superficial	One thin area of deep blue staining with tidemark unclear	Disorganized
**Pannus present**	No	—	—	Yes

**Table 2:** Interobserver agreement.

Sheep n°	ICC (95% CI 0.95–0.99)
1	0.80934561
2	0.97668054
3	0.87859618
4	0.92000267
5	0.90783898
6	0.94328601
7	0.94706078
8	0.97789721

## Data Availability

The dataset supporting the conclusions of this article is included within the article.

## Abbreviations

AC	Articular cartilage
ACL	anterior cruciate ligament
AIG	anterior-inferior glenoid
AIH	anterior-inferior humerus
ASG	anterior-superior glenoid
ASH	anterior-superior humerus
BML	bone marrow lesions
CSA	cartilage surface area
CTL	cervical, thoracic, lumbar
EDTA	Ethylenediaminetetraacetic acid
GH	glenohumeral
ICRS	International Cartilage Regeneration and Joint Preservation Society
MRI	Magnetic Resonance Imaging
MSAS	Murine Shoulder Arthritis Score
OA	Osteoarthritis
OARSI	Osteoarthritis Research Society International
OSAS	Ovine Shoulder Arthritis Score
PIG	posterior-inferior glenoid
PIH	posterior-inferior humerus
PSG	posterior-superior glenoid
PSH	posterior-superior humerus
SD	standard deviation
sMOASS	Sheep MRI Osteoarthritis Shoulder Score
